# A Clinical Diagnostic System for Late-Stage Neuropsychiatric Lyme Borreliosis Based upon an Analysis of 100 Patients

**DOI:** 10.3390/healthcare8010013

**Published:** 2020-01-06

**Authors:** Robert C. Bransfield, Dylan M. Aidlen, Michael J. Cook, Sagar Javia

**Affiliations:** 1 Department of Psychiatry, Rutgers-Robert Wood Johnson Medical School, Piscataway, NJ 08854, USA; 2 Department of Biology, Northeastern University, Boston, MA 02115, USA; dylanaidlen@gmail.com; 3 Independent Researcher, Dorset BH23 5BN, UK; mcook98@msn.com; 4 Independent Researcher, Easton, PA 18045, USA; sjavia@gmail.com

**Keywords:** Lyme disease, clinical assessment, chronic Lyme disease, late-stage Lyme disease, neuropsychiatric Lyme disease, *Borrelia burgdorferi*, Lyme borreliosis, psychiatric, Lyme clinical assessment

## Abstract

Many late-stage chronic Lyme disease clinical findings are neuropsychiatric. A total clinical assessment is critical in diagnosis, especially since controversy surrounds the reliability of laboratory testing. The clinical findings of one hundred Lyme disease patients with chronic neuropsychiatric symptoms were entered into a database. The prevalence of each clinical finding pre-infection and post-infection was compared and calculated within the 95% confidence interval. Patients had minimal symptoms pre-infection, but a high post-infection prevalence of a broad spectrum of acquired multisystem symptoms. These findings included impairments of attention span, memory, processing, executive functioning, emotional functioning, behavior, psychiatric syndromes, vegetative functioning, neurological, musculoskeletal, cardiovascular, upper respiratory, dental, pulmonary, gastrointestinal, genitourinary, and other symptoms. The most prevalent symptoms included sustained attention impairments, brain fog, unfocused concentration, joint symptoms, distraction by frustration, depression, working memory impairments, decreased school/job performance, recent memory impairments, difficulty prioritizing multiple tasks, fatigue, non-restorative sleep, multitasking difficulties, sudden mood swings, hypersomnia, mental apathy, decreased social functioning, insomnia, tingling, word finding difficulties, name retrieval, headaches, sound hypersensitivity, paresis, anhedonia, depersonalization, cold intolerance, body temperature fluctuations, light sensitivity and dysfluent speech. The average patient had five symptoms pre-infection and 82 post-infection. Pattern recognition is critical in making a diagnosis. This study was used to develop three clinical assessment forms.

## 1. Introduction

### 1.1. Historical Perspective

Lyme borreliosis, also called Lyme disease, is a tick-borne disease caused by an infection with *Borrelia burgdorferi sensu lato* complex, a spirochete that is more complex and more difficult to treat than syphilis [1,2]. Other tick-borne diseases and opportunistic infections may accompany the *Borrelia* infection and contribute to a complex interactive infectious process [3]. It has been called by many different names throughout the past 100 years including acrodermatitis chronicum atrophicans in Europe for a characteristic late-stage rash. Lyme disease was at one time called Lyme arthritis and early clinical diagnostic criteria focused heavily upon the erythema migrans rash, migratory arthralgia, arthritis, and Bell’s palsy. A large number of symptoms associated with Lyme disease have been documented, however there is wide variability of specific symptoms present in a given patient. This makes it difficult to establish clearly defined diagnostic criteria, especially for late-stage disease. A number of definitions for the late-stage, chronic, manifestations have been proposed [4,5,6]. Like syphilis, the symptoms that occur later in the course of the illness are different from the early symptoms. It is well recognized that some patients with Lyme disease have persistent, late-stage, chronic neuropsychiatric symptoms [7,8,9]. Recognizing the full spectrum of these symptoms and quantitating the severity of these symptoms are major challenges. It is difficult to measure a disease when laboratory tests have significant limitations and clinical presentations can be highly variable [10,11,12,13]. These limitations compromise the accuracy of both diagnosis and the measurement of response in clinical treatment and vaccine studies.

### 1.2. Assessment, Total Clinical Assessment or Laboratory Assessment?

A tradition in mainstream medicine is to first perform a thorough clinical exam, to then consider lab tests when they might help with the diagnosis, and to use clinical judgment to develop an individualized diagnosis and treatment plan. We treat patients, not diseases [14]. An individualized approach is particularly significant when dealing with complex and poorly understood multisystem diseases. In opposition to this conservative and traditional approach, some have attempted to oversimplify the diagnosis of Lyme disease by reducing the diagnosis to reliance upon the United States Centers for Disease Control and Prevention (CDC) surveillance definition [15]. However, the surveillance definition has never been intended to be a sole diagnostic criterion, particularly in late-stage disease. Although meeting the surveillance definition for Lyme disease may confirm the diagnosis, not meeting the surveillance definition does not rule out the diagnosis of the disease. This has been emphasized by the Centers for Disease Control and Prevention and has been supported by studies performed by them [16,17]. In spite of this warning, many physicians fail to perform adequate clinical examinations when suspecting Lyme disease, and by default place excessive reliance upon laboratory testing that can be highly flawed [10,11,12,13].

The excessive reliance upon laboratory testing, particularly when dealing with the chronic, late-stage manifestations results in considerable controversy. The most commonly used laboratory testing has never been standardized for late-stage disease, and the commonly used antibody detection methods are of questionable value when testing for a microbe that evades and suppresses the immune system [18].

A total clinical assessment is a diagnostic standard of care throughout medicine, and there is no reason why Lyme disease should be an exception. The chronic, late-stage clinical findings are associated with a broad spectrum of neuropsychiatric and other multisystem symptoms. Although some diagnoses can be made with specific signs and symptoms, other conditions instead require a recognition of symptom patterns and disease progression patterns. Since no two patients with late-stage manifestations of Lyme disease show exactly same symptoms, establishing diagnostic clinical criteria is challenging. A structured clinical assessment has previously been described for the diagnosis and assessment of patients when their screening suggests Lyme disease should be considered in the differential diagnosis [9]. Several studies have addressed the prevalence of different symptoms associated with neuropsychiatric Lyme disease [9,19,20,21,22,23,24,25,26,27,28,29,30,31]. Two prior studies looked at the prevalence of clinical findings pre-infection and post-infection. However, these studies focused on Lyme disease patients who were suicidal and aggressive [32,33]. Although several studies describing clinical findings were performed previously, no prior study looked at the broad spectrum of clinical findings associated with chronic, late-stage, neuropsychiatric Lyme disease and compared these findings to the baseline state of health. Recording the type of clinical findings and documenting the prevalence of these findings may assist in establishing a reference point of pattern recognition when performing an overall clinical assessment when a clinician is suspecting a diagnosis of late-stage neuropsychiatric Lyme disease. Such a clinical assessment system would be useful in diagnosing these patients, establishing an individualized treatment plan, and assessing treatment response.

### 1.3. Clinical Assessment Scales and Treatment Studies

Lyme disease treatment studies that are frequently referenced use clinical assessment scales that were not specifically developed for Lyme disease. For example, both the Berende and the Klempner studies used the Health Status Inventory Short Form to measure treatment effectiveness [34,35]. However, this scale is a general level of functioning scale, highly subjective, and it was not designed or standardized to measure level of functioning for Lyme disease [36]. Other treatment studies only measured specific symptoms. The primary outcome in the Fallon study was neurocognitive performance, specifically, memory [37]. The primary clinical outcomes measured in the Krupp study was fatigue, measured by the Fatigue Severity Scale and cognitive functioning (mental speed) [38]. When an assessment that does not fully measure the symptoms associated with Lyme disease are used in a study, it may result in inaccurate results. A diagnostic system is needed that fits with the full clinical spectrum of symptoms seen in late-stage Lyme disease.

## 2. Materials and Methods

The objective of this study is to describe the clinical presentation of Lyme borreliosis patients with chronic, late-stage, psychiatric symptoms from a retrospective review of 100 charts, and to develop a clinical assessment system from these findings.

The first author specializes in working with treatment-resistant psychiatric illnesses. Some of these treatment resistant patients had psychiatric symptoms associated with Lyme disease. The first author initially developed the assessment form used in this study over 20 years ago and has used this assessment to evaluate a few thousand patients when late-stage Lyme disease was part of the differential diagnosis. The same assessment was performed on all patients by the first author. In the process of performing the assessment, the date of infection was first established. The presence of each clinical finding was established during the examination at the baseline before infection (pre-infection), the emergence of the clinical finding since becoming infected, and the presence of the clinical finding at the time of the assessment. All assessments on each clinical finding were personally performed by the first author, who is a board-certified psychiatrist. No information on these patients was obtained from self-reported check lists. Family members and/or significant others were often present during the evaluation and their participation often helped improve the accuracy of the assessment. When a clinical finding was fully present, a (+) was entered in the assessment form. When a clinical finding was partially present, a (+/−) was entered in the assessment form. If a clinical finding was only slightly present, a (sl) was entered on the assessment form. When a clinical finding was absent, a (−) was entered in the assessment form.

Inactive charts were reviewed to search for charts in which the full assessment was performed documenting psychiatric symptoms associated with Lyme disease. All patients in this study resided in the Continental United States and appear to have been infected in the Continental United States. Since Lyme disease impacts all age groups no age exclusionary criteria were used in this study, with the exception that no congenital Lyme disease cases were included in this study. Inclusion criteria for this study were charts documenting psychiatric findings in patients who had met the Centers for Disease Control and Prevention surveillance definition for Lyme disease. These criteria included erythema migrans rash, nervous system criteria, musculoskeletal criteria, cardiovascular criteria and/or laboratory criteria and most patients met more than one criterion. More specifically, 100% met neurological criteria with cranial nerve and other neurological findings, 81% met the musculoskeletal criteria, 52% met the criteria based upon erythema migrans rash, and 11% met cardiac criteria with heart block. In addition, 100% met laboratory criteria with all having positive Lyme Western blots, some on multiple testing, some were also positive with spinal fluid testing and polymerase chain reaction testing for DNA. Only testing from laboratories validated by the Clinical Laboratory Improvement Amendments of the United States Department of Health and Human Services Centers for Medicare and Medicaid Services was considered valid. Charts in which the diagnosis of Lyme disease was unclear or where the diagnosis of Lyme disease was based upon total clinical criteria, but not meeting Centers for Disease Control and Prevention surveillance criteria were excluded from this study.

Names were converted to an identifier before entering the findings into aggregate data. The aggregate data of baseline health status for each clinical finding before infection served as a control for each clinical finding. The aggregate data of pre-infection and post-infection findings was compared. In addition, the clinical findings assessed included age, sex, documented history of whether there was exposure to an area endemic to Lyme disease, a tick bite, the presence of erythema migrans rash, a flu-like illness, and a reoccurrence of an erythema migrans rash and the length of time between infection and initial antibiotic treatment. The clinical findings were organized into the following categories—attention span; memory; processing; executive functioning; imagery; emotional; behavioral; psychiatric syndromes; vegetative functioning (energy, sleep, eating, sex, temperature control); neurological (headaches, cranial nerves, seizures, other); musculoskeletal; cardiovascular; upper respiratory, dental and pulmonary; gastrointestinal; genitourinary; other; and symptom patterns.

The total impairments of all the 100 patients analyzed was recorded pre-infection and post-infection. When a clinical finding was fully present, it was entered into the database as (1). If a clinical finding was partially present (+/−), it was entered as (0.5). If a clinical finding was only slightly present (sl), it was entered into the database as (0.25). If a clinical finding was not present (−), it was entered as (0). When a clinical finding was not relevant or not addressed in 100% of the patients, the percent was calculated accordingly. The number of patients demonstrating each clinical finding was added and the 95% confidence interval was calculated to determine the diagnostic relevance for each clinical finding.

Since the 100 patients studied or their family members present at the evaluation might have difficulty correctly remembering their pre-infection health status, two secondary control groups were established to assess the validity of the primary control group. The two secondary control groups include the United States National Comorbidity Survey Replication of the prevalence of 12 month Diagnostic and Statistical Manual-IV mental disorders, and the same assessment on heathy medical students who do not have Lyme disease [39].

The post-infection clinical findings were then compared to two other groups. One group was an age-matched group of patients who were evaluated with the Lyme disease assessment but were found to have some other condition. The other group was a comparison to post-infection clinical findings that were seen in a review of other studies [9].

Clinical assessment forms were then created that a clinician can download from this article and use when assessing the possibility of chronic, late-stage, neuropsychiatric Lyme borreliosis. Some clinical findings are added to the list for future reference that contains no data in this retrospective study. These are clinical findings the first author has seen in some Lyme disease patients, but the prevalence was not analyzed in this chart review. The forms provided include three forms—a common symptom 61-item form, a 24-item pre-evaluation form and a full assessment. The common symptom 61-item form includes all the clinical findings that are present in ≥50% of the patients. The short form consists of 24 questions that reduces the information collection but is faster and easier for patients to complete in an unsupervised environment, for example prior to the clinician visit. The questions were selected based on the presence of symptoms in ≥50% of more of study patients and it only included question considered to be understandable to the general public. The full assessment is more thorough and includes all the clinical findings found to be statistically significant and clinically significant, as well as some clinical findings that appear to be clinically significant but have no database for comparison.

### Ethical Considerations

The Hackensack Meridian Health Institutional Review Board, Neptune, NJ, USA, approved this study (IRB # 201704192J). Patient consent to review their medical records was not required by the Institutional Review Board as there was minimal risk to subjects, no subject identifiers or links to identifiers were used or collected, and it was a retrospective chart review of already existing data.

## 3. Results

### 3.1. Results, Overview

Among the 100 charts reviewed, the average age was 38, with the youngest being 6 years old and the oldest being 89 years old. There were 41 males and 59 females. There were 85 between 18 and 65 years old; 5 older than 65, including 3 who were older than 69; and 10 below 18, including 3 who were below age 13. All in this study appear to have been infected in the United States. Ethnicity and race were not recorded. There was a history of exposure to an endemic area in 98%, a history of tick bite(s) in 60%, an erythema migrans rash in 53%, early flu-like symptoms in 68%, and a recurrent erythema migrans rash in 37%. All met United States Centers for Disease and Prevention surveillance definition at some point in the course of their illness. There were 30 who were diagnosed and treated within 6 months of infection but continued to have disease progression and the development of chronic, late-stage clinical findings. The 70 others had a delayed diagnosis and treatment, with the average delay being 9 years. The longest delay between infection and treatment was 40 years. The average age at the time of infection was 30 years old.

The average patient had five (4.59) clinical findings pre-infection and 82 (82.02) clinical findings post-infection. Most clinical findings evaluated showed a statistically significant difference when comparing the prevalence of these clinical findings pre-infection to the prevalence of the same clinical findings post-infection. There were some clinical findings that showed an increase when comparing the prevalence pre-infection to the prevalence post-infection, but not a statically significant increase. These clinical findings include intrusive sexual images, homicidality, posttraumatic stress disorder, papilledema, iritis, uveitis, optic neuritis, grand mal seizures, partial seizures, Tourette’s, torticollis, periostitis, pericarditis, murmur, hypertensive crisis, gastroparesis, hepatitis, pancreatitis, gall stones, inflammatory bowel, interstitial cystitis, acrodermatitis chronicum atrophicans, and lymphocytoma. The only clinical findings assessed that showed no increase post-infection were spasticity and erythema of the palms and soles.

### 3.2. Statistically Significant Clinical Findings

Clinical findings comparing pre-infection health to post-infection health with the 95% confidence intervals demonstrated a statistical difference for multiple sign and symptoms and are shown in Table 1.

Statistically significant impairments were cognitive, imagery, emotional, psychiatric syndromes, vegetative impairments (energy, sleep, eating, sexual functioning and temperature control), neurological (headaches, cranial nerve, and other), musculoskeletal, cardiovascular, upper respiratory, dental and pulmonary, gastrointestinal, genitourinary and other clinical findings.

The following are a list of impairments demonstrating a statistically significant difference when comparing pre-infection status to post-infection status within the 95% confidence interval.

- Cognitive impairments include impairments of attention, memory, processing and executive functioning. Impairments of attention include impaired sustained attention and allocation of attention, distraction by frustration, hypersensitivity to sound, hypersensitivity to light, hypersensitivity to touch and hypersensitivity to smell. Processing impairments include letter reversals, spelling errors, word substitution errors, number reversals, spatial perceptual distortions, optic ataxia, left–right confusion, and impairments of reading comprehension, auditory comprehension, sound localization, transposition of laterality, calculation, fluency of speech, fluency of written language and handwriting. Executive functioning impairments include unfocused concentration, brain fog, racing thoughts, obsessive thoughts, mental apathy and difficulty with multitasking and abstract reasoning.
- Imagery impairments include impaired capacity for visual imagery, intrusive aggressive images, other intrusive images, hypnagogic hallucinations, vivid nightmares, illusions, and hallucinations (auditory, visual, olfactory, and tactile).
- Emotional impairments include decreased frustration tolerance, sudden mood swings, paranoia, crying spells and anhedonia.
- Dissociative symptoms include depersonalization, derealization and dissociative episodes.
- Behavioral symptoms include disinhibition, exaggerated startle reflex, suicidality, accident proneness, decreased social functioning, decreased job/school performance, marital/family problems, substance abuse, legal difficulties, compensatory compulsions and dropping objects from their hands.
- Psychiatric syndromes include depression, rapid cycling bipolar, panic disorder, obsessive compulsive, social anxiety disorder, social anxiety disorder and generalized anxiety disorder.
- Sleep impairments include non-restorative sleep, early insomnia, mid insomnia, late insomnia, hypersomnia, loss of circadian rhythm and hypersomnia.
- Eating impairments include anorexia, weight loss, non-appetite overeating, weight gain without increased food intake and weight gain with increased food intake.
- Sexual impairments include decreased libido, increased libido, decreased arousal, decreased orgasm, and menstrual irregularity.
- Temperature control impairments include body temperature fluctuations, skin flushing, intolerance to heat, intolerance to cold and decreased body temperature.
- Headaches include cervical radiculopathy, migraine, coital cephalgia, temporal mandibular joint, tension, cluster and sinus headaches. Cranial nerve impairments include all cranial nerves, including multiple ophthalmologic symptoms.
- Other neurological findings include sensory neuropathy symptoms (numbness, tingling, sensory loss, burning, static electricity sensation, formication and stabbing sensation), paresis, tremors, twitching, muscle tightness, restless leg, myoclonic jerks, herniated discs and positive Romberg testing.
- Musculoskeletal findings include joint (pain, swelling, tightness, crepitations), bone thinning/fractures, epicondylitis, plantar fasciitis, fibromyalgia, myalgia, chondritis, tendonitis and carpal tunnel syndrome.
- Cardiac findings include chest pain, heart block, mitral valve prolapse, racing pulse, episodes of rapid and slow heart rate, postural orthostatic hypotension, cardiomyopathy and hypertension.
- Upper respiratory, dental and pulmonary findings include swollen glands, cough, allergies, nose bleeds, tooth pain, periodontal disease, shortness of breath and asthma.
- Gastrointestinal findings include upper gastrointestinal distress, irritable bowel, abdominal bloating, inflammatory bowel and cholecystitis.
- Genitourinary findings include genital pain, breast tenderness, lactation, menstrual irregularity, urinary incontinence, spastic bladder, recurrent urinary infections, anesthesia of genitalia and atrophy of genitalia.
- Other findings include chronic pain, alcohol intolerance, multiple chemical sensitivity, hair loss, thyroid dysfunction, Wilson syndrome, adrenal insufficiency, hypoglycemia, vasculitis, ankle edema, splenomegaly, ecchymosis, lymphocytoma and erythema of palms or soles.
- Symptom patterns include Herxheimer reactions, progression of symptoms, fluctuation of symptoms, stress increases symptoms, and antibiotics reduce symptoms.

### 3.3. Most Prevalent Pre-Infection Clinical Findings

The most prevalent clinical findings pre-infection were calculation difficulties, 10%; depression, 9%; spelling errors, 8%; poor handwriting, 8%; sustained attention impairments, 7%; distracted by frustration, 7%; social anxiety disorder, 7%; murmur, 7%; allergies, 7%; difficulty allocating attention adequately, 6%; name retrieval, 6%; reading comprehension impairments, 6%; left–right confusion 6%; difficulty prioritizing multiple tasks, 6%; diminished social functioning, 6%; posttraumatic stress disorder, 6%; upper gastrointestinal distress, 6%; irritable bowel, 6%; recent memory impairments, 5%; word substitution errors, 5%; auditory comprehension impairments, 5%; decreased frustration tolerance, 5%; initial insomnia, 5%; loss of circadian rhythm, 5%; decreased body temperature, 5%; sinus headaches, 5% and restless leg, 5%.

#### 3.3.1. Validating the Pre-Infection Control Group with Other Control Groups

Two secondary healthy control groups contributed to assessing the validity of the primary pre-infection control group. The results of the United States National Comorbidity Survey Replication of the prevalence of 12 month Diagnostic and Statistical Manual-IV mental disorders was used to contribute to the validation of the prevalence of pre-infection clinical findings, and a survey of healthy medical students was used to contribute to validating the number of pre-infection clinical findings seen in a healthy control group.

##### Comparison of the Pre-Infection Control Group to the National Comorbidity Survey

The United States National Comorbidity Survey Replication of the prevalence of 12 month Diagnostic and Statistical Manual-IV mental disorders assesses the prevalence of some of the mental disorders that were also calculated in the pre-infection baseline [39]. Disorders included in both the Comorbidity Study and the Lyme assessment include panic disorder, obsessive compulsive disorder, social anxiety disorder, generalized anxiety disorder and posttraumatic disorder. Since dysthymia (1.5%) and major depression (6.7%) were calculated separately in the National Comorbidity Survey and together in the Lyme disease survey, these two statistics were added together. Also, intermittent explosive disorder was in the National Comorbidity Survey, which was comparable to explosive anger in the Lyme disease survey. The close correlation between the two surveys contributes to the validation of the accuracy of the pre-infection control group. The comparison between the Lyme pre-infection control group and the National Comorbidity Survey is shown in Table 2.

##### Comparison of the Pre-Infection Control Group to a Healthy Control Group

To help validate the baseline of the average subject before infection, records were retrieved where the assessment had been administered anonymously to 23 healthy medical students during an educational program on Lyme disease which discussed the use of this assessment form for the evaluation of Lyme disease. Any record showing a history of Lyme disease was not included in the data. The average age of the participants was 24. There were 16 with no history of tick bites and seven with a history of tick bites. There were 22 with no history of a bull’s eye rash and one with a history of a bull’s eye rash. The average number of clinical findings in this group was four. The greatest number of clinical findings was 13 and the least was 0.

### 3.4. Most Prevalent Post-Infection Clinical Findings

The most prevalent clinical findings post-infection in this study group that were greater than or equal to 50% were sustained attention impairments, 84%; brain fog, 84%; unfocused concentration, 81%; joint symptoms, 81%; distracted by frustration, 79%; depression, 79%; working memory impairments, 78%; decreased school/job performance, 78%; recent memory impairments, 77%; difficulty prioritizing multiple tasks, 76%; fatigue, 76%; non-restorative sleep, 76%; multitasking difficulties, 74%; sudden mood swings, 74%; hypersomnia, 73%; mental apathy, 72%; decreased social functioning, 72%; insomnia, middle, 72%; tingling, 71%; word finding difficulties, 70%; initial insomnia, 70%; name retrieval, 68%; headaches, 68%; sound hypersensitivity, 66%; paresis, 66%; anhedonia, 64%; depersonalization, 64%; cold intolerance, 64%; body temperature fluctuations, 63%; sensitivity to bright light, 63%; dysfluent speech, 62%; decreased libido, 60%; night sweats, 60%; reading comprehension difficulties, 59%; chills, 59%; numbness, 59%; compensatory compulsions, 58%; insomnia, late, 58%; heat intolerance, 58%; tension headaches, 57%; spelling errors, 56%; obsessive thoughts, 56%; floaters, 56%; twitching, 56%; muscle tightness, 56%; word substitution errors, 55%; racing thoughts, 54%; myalgia, 54%; generalized anxiety disorder, 53%; dizziness, 53%; number retrieval impairments, 52%; dropping objects, 52%; decreased body temperature, 52%; optic ataxia, 51%; calculation impairments, 51%; abstract reasoning impairments, 51%; tinnitus, 51%; crying spells, 50%; blurred vision, 50%; restless leg, 50%; and irritable bowel, 50%. These are shown in Table 3.

### 3.5. Comparison of Post-Infection Clinical Findings

In the database, 10 charts, which represented an age-matched control group, were reviewed. These patients were assessed for the possibility of Lyme disease, but were diagnosed with conditions other than Lyme disease. In this group, the average age was 33 years old. The age range was 7 years old to 73 years old. The average age was 33 years old. The average number of clinical findings in this group was 22 (21.7).

### 3.6. Comparison of Post-Infection Clinical Findings to Other Studies

The post-infection findings of this study can be compared to the findings seen in other groups of Lyme disease patients that have previously been published in 20 other studies of Lyme disease that have included a total of 23 different patient groups. The groups with the most severe symptoms were the Lyme disease patients who were homicidal, followed by the patients who were suicidal [9]. Table 4 compares the post-infection clinical findings to the prevalence of the same findings in other studies.

## 4. Discussion

It is recognized these patients were seeking treatment by a psychiatrist and therefore there was probably a selection bias of Lyme disease patients who had a greater number of psychiatric manifestations. Therefore, this assessment is particularly useful when evaluating patient who may have neuropsychiatric symptoms and there is the suspicion that these symptoms may have been caused by Lyme disease. This study helps to demonstrate the broad spectrum of neuropsychiatric and other symptoms that are seen as late-stage manifestations of Lyme disease. The results of this study are a database to develop assessment tools that can be used in the assessment of patients when the diagnosis of Lyme disease is part of the differential diagnosis. The assessment of Lyme disease can begin with some screening questions which consist of the following [Supplementary Materials] [9]:

- Do you live, vacation, or engage in occupational or other activities in areas that may expose you to ticks?
- Have family members, neighbors, or the family dog been infected?
- Is there a history of a tick bite, possibly with a flu-like illness and/or a bull’s eye or other rash?
- Is there a point at which your health declined, followed by a fluctuating progression and development of multi-systemic symptoms, including cognitive, psychiatric, neurological, and somatic symptoms adversely impacting school, social life, family life?
- Have you ever been treated for Lyme disease, suspected you had Lyme disease but was told it was ruled out?
- Have antibiotics ever caused a sudden worsening followed by an improvement of symptoms?”

If the screening assessment provides diagnostic suspicion, a more thorough assessment or a full assessment can be performed.

One type of assessment is the common symptom 61-item assessment, which includes clinical findings which were present in ≥50% of the patients in the database of this article and is shown in Table 5.

Another option, which may be used more for pre-evaluation, is the 24-item assessment that is shown in Table 6. 

A third more comprehensive option is the full clinical assessment that is shown in Table 7. 

The more thorough assessment includes an assessment for the presence of all the clinical findings evaluated which are more prevalent in these patients, including those which did not reach statistical significance. It adds greater specificity and support to the diagnosis of the late-stage manifestations of Lyme disease. 

The only symptoms totally specific to Lyme disease seen in these patients are erythema migrans rash and acrodermatitis chronicum atrophicans. However, a common pattern with these patients may include early erythema migrans rash, flu-like symptoms and cranial nerve symptoms, followed by a combination of musculoskeletal symptoms, fatigue and cognitive impairments and the later appearance of other multisystem symptoms that may be psychiatric, neurological, cardiovascular, gastrointestinal, genitourinary, upper respiratory, dental, pulmonary, genitourinary and other symptoms. These symptoms and clinical findings can progressively expand and increase in severity over time. When a patient develops such a diversity of symptoms with an expanding number and intensity, one explanation can be one condition occurring with multisystem manifestations. Another possible explanation is a few concurrent conditions may have occurred at the same time. Since the pathophysiology and causal association between Lyme disease and many of these symptoms has previously been explained, a causal association between Lyme disease and the symptoms these patients experienced is a likely explanation [9,40,41,42,43,44,45]. Also, since the onset of the symptoms was associated with a tick bite, a bull’s eye rash and positive Lyme serology in these patients, this adds further support that the most likely explanation is the symptoms were caused by Lyme disease. However, a possible contributory role from other tick-borne, other coinfections, or other coexisting conditions can also be considered [46].

The differential diagnosis of Lyme borreliosis vs. other medical conditions is complex. Lyme, like syphilis can appear as many different conditions. For this reason, it has been called the “new great imitator” [28]. Any condition can coexist with Lyme disease. Therefore, the diagnosis of some other condition, alone, does not rule out the diagnosis of Lyme disease, and the diagnosis of Lyme disease does not rule out the diagnosis of some other comorbid condition. The question in diagnosis is what diagnosis can explain the symptoms seen in any given patient? There are other conditions that cause multisystem illnesses, such as toxicities, deficiencies, other systemic infections and immune disorders that should be considered in the differential diagnosis. A comprehensive examination like the one described in this article can also be useful in identifying symptom patterns seen in other illnesses. The common differential diagnosis that is considered for late-stage Lyme disease include myalgic encephalitis/chronic fatigue syndrome, fibromyalgia, and psychosomatic illness. Myalgic encephalitis/chronic fatigue syndrome has a more acute onset and also has dysautonomia and does not include the neurological and arthritic symptoms seen in Lyme disease. It also has different, but some overlapping findings in spinal fluid [47]. Fibromyalgia can occur following Lyme disease, but also occur in the absence of Lyme disease [22,48,49]. Psychosomatic illness is usually not a multisystem illness with such a diversity of symptoms [50]. In the differential diagnosis, pattern recognition is critical. The greater the number of multisystem symptoms that are associated with Lyme, the greater the likelihood that Lyme disease is the diagnosis. A coinfection screen can be performed when considering the presence of coinfections [Supplementary Materials]. It is difficult to explain this unique combination of clinical findings on the basis of some other diagnosis. When the first author performed this assessment on patients who did not have Lyme disease but had some other condition, there was a lesser number and a different pattern of clinical findings that were seen. A more comprehensive exam, such as the one described, improves the likelihood of a more accurate diagnosis, whether it be Lyme disease or some other diagnosis. Laboratory findings may also be considered in making the diagnosis, while being aware of some of the limitations of current laboratory testing when evaluating late-stage disease.

Based upon the findings of this study, clinical assessment forms can be used by clinicians when assessing a possible case of late-stage neuropsychiatric Lyme disease and to document clinical status following treatment (Table 5, Table 6 and Table 7 and Screening Assessment and Coinfection Screen in Supplementary Materials).

After completing the assessment(s), it is also important to consider any differential diagnosis that would better explain the pattern of symptoms seen in the patient. Lyme disease is the most likely diagnosis if the pattern of clinical findings and course of illness is most compatible with a diagnosis of Lyme disease compared to other conditions in the differential diagnosis.

After the diagnosis is made, the next part of the assessment is to consider which clinical findings are most severe and most significant in contributing to disease perpetuation and progression and to then prioritize these findings in order of significance. When these symptoms are prioritized in this manner, it helps in planning the symptomatic treatment of these patients. The symptom priority may change as symptoms improve from treatment.

This article is a basic introduction to a comprehensive clinical assessment. Further study, field testing, comparing the results of these assessments on patients with other diagnoses, and independent validation by others shall help to confirm these findings and shall be useful to develop objective evaluations to help standardize this assessment. Two other clinical assessment forms currently exist, The Burrascano Checklist of Current Symptoms and the Horowitz Multiple Systemic Infectious Disease Syndrome Assessment [3,51]. Compared to the other two assessment systems, this assessment is more targeted towards neuropsychiatric symptoms, assesses a broader spectrum of multisystem symptoms, and the full assessment requires more time and clinical expertise on the part of the evaluator.

The physician assessment forms can be scored in a quantitative manner by using the Clinical Global Impression Scale (CGI), a scale that is used in many United States Federal Drug Administration studies [52]. The CGI-Severity Scale (CGI-S) is rated as the average severity level in the prior week as 1 = normal, not at all ill; 2 = borderline ill; 3 = mildly ill; 4 = moderately ill; 5 = markedly ill; 6 = severely ill; 7 = among the most extremely ill patients. Response to treatment can be measured by the CGI-Improvement Scale (CGI-I). The CGI-Improvement Scale (CGI-I) compares the patient’s average clinical change in the prior week in the baseline status since the initiation of treatment. It is rated as “1 = very much improved since the initiation of treatment; 2 = much improved; 3 = minimally improved; 4 = no change from baseline (the initiation of treatment); 5 = minimally worse; 6 = much worse; 7 = very much worse since the initiation of treatment” [52].

## 5. Conclusions

The prevalence of psychiatric and other symptoms seen in 100 patients with late-stage Lyme neuroborreliosis was compared pre-infection vs. post-infection and the confidence intervals were calculated. The validity of pre-infection health status was partially confirmed by comparing it to two additional groups. Also, the post-infection findings were compared to patients with other systemic illnesses and compared to results from other studies. The patients in this study had minimal symptoms pre-infection (average of five), but a high post-infection prevalence of a broad spectrum of acquired multisystem symptoms, including neuropsychiatric symptoms, after acquiring Lyme borreliosis (average of 82). These findings included impairments of attention span, memory, processing, executive functioning, emotional functioning, behavior, psychiatric syndromes, vegetative functioning, neurological, musculoskeletal, cardiovascular, upper respiratory, dental, pulmonary, gastrointestinal, genitourinary, and other symptoms. The most prevalent symptoms included sustained attention impairments, brain fog, unfocused concentration, joint symptoms, distraction from frustration, depression, working memory impairments, decreased school/job performance, recent memory impairments, difficulty prioritizing multiple tasks, fatigue, non-restorative sleep, multitasking difficulties, sudden mood swings, hypersomnia, mental apathy, decreased social functioning, insomnia, tingling, word finding difficulties, name retrieval, headaches, sound hypersensitivity, paresis, anhedonia, depersonalization, cold intolerance, body temperature fluctuations, light sensitivity and dysfluent speech. Since this study included Continental United States patients, other symptom patterns may be seen in other geographical areas. In this study, there was a large separation between the average number of clinical findings pre-infection (5) and in in healthy controls (4) vs. other diagnoses (22) vs. post-infection (82). All of the patients with Lyme disease had multisystem symptoms. The greater number of multisystem symptoms correlated with a diagnosis of Lyme disease, and a lesser number of multisystem symptoms correlated with not having a diagnosis of Lyme disease. The results of this study were then used to develop three clinical assessment forms that can be used when the diagnosis of Lyme disease is suspected. This includes the 24-item patient pre-evaluation form, the common symptom 61-item assessment, and the full assessment. If the results of this study are then generalized to other patients, it suggests a greater number of multisystem symptoms correlates with the possibility of a diagnosis of Lyme, and a lesser number of multisystem symptoms correlates with a lower possibility of Lyme disease. The number of clinical findings cannot be rigidly implemented as a diagnostic criterion for a number of reasons. These reasons include: (1) some symptoms are more non-specific and prevalent while other symptoms are more specific; (2) some symptoms may be caused by some other condition; (3) Lyme borreliosis can be latent and not currently symptomatic; (4) Lyme borreliosis can sometimes have a unique presentation; and (5) individualized clinical judgment is always needed. The assessment, however, is a tool that clinicians can use to acquire information to look for pattern recognition. When combined with clinical judgment this assessment can help improve clinical diagnostic effectiveness. Since controversy surrounds the commonly used laboratory testing for Lyme disease, the use of clinical diagnostic assessments can be of value in considering a diagnosis of Lyme borreliosis. The assessment systems can also be used to track further disease progression, improvement or response to treatment. The use of this assessment, further interpretation of the data, further validation, and/or refinement by others can help to further develop these clinical assessment systems for the clinical diagnosis of Lyme borreliosis.

## 6. Patents

None of the authors report having any patents that are relevant to this article.

## Figures and Tables

**Table 1:** The full assessment list includes clinical findings that are statistically significant pre- vs.- post-infection, not statistically significant pre- vs. post-infection and without statistical data.

Clinical Impairment	Pre-Infection	95% CI	Post-Infection	95% CI
**Attention span**				
Sustained attention	7%	(2–12%)	84%	(77–91%)
Distracted by frustration	7%	(2–2%)	79%	(71–87%)
Allocation of attention	6%	(1–11%)	66%	(57–75%)
Hypersensitivity to sound	3%	(0–6%)	66%	(57–75%)
Hypersensitivity to light	2%	(0–5%)	63%	(54–72%)
Hypersensitivity to touch	2%	(0–5%)	41%	(31–51%)
Hypersensitivity to smell	5%	(1–9%)	36%	(27–45%)
Sensory overload	No data			
**Memory**				
Working memory	3%	(0–6%)	78%	(70–86%)
Recent memory	5%	(1–9%)	77%	(69–85%)
Working spatial memory	1%	(0–3%)	46%	(36–56%)
Remote memory	4%	(0–8%)	35%	(26–44%)
**Memory retrieval**				
Words	3%	(0–6%)	70%	(61–79%)
Names	6%	(1–11%)	68%	(59–77%)
Numbers	3%	(0–6%)	52%	(42–62%)
Geographical/spatial	1%	(0–3%)	49%	(39–59%)
Faces	1%	(0–3%)	23%	(15–31%)
Motor memory	1%	(0–3%)	10%	(4–16%)
**Processing**				
Fluency of speech	4%	(0–8%)	62%	(52–72%)
Reading comprehension	6%	(1–11%)	59%	(49–69%)
Spelling errors	8%	(3–13%)	56%	(46–66%)
Word substitution errors	5%	(1–9%)	55%	(45–65%)
Calculation	10%	(4–16%)	51%	(41–61%)
Optic ataxia	1%	(0–3%)	51%	(41–61%)
Auditory comprehension	5%	(1–9%)	49%	(39–59%)
Handwriting	8%	(3–13%)	47%	(37–57%)
Letter reversals	2%	(0–5%)	45%	(35–55%)
Fluency of written language	2%	(0–5%)	43%	(33–53%)
Number reversals	1%	(0–3%)	39%	(29–49%)
Left–right confusion	6%	(1–11%)	30%	(21–39%)
Transposition of laterality	2%	(0–5%)	22%	(14–30%)
Spatial perceptual distortions	1%	(0–3%)	21%	(13–29%)
Sound localization	3%	(0–6%)	19%	(11–27%)
**Executive functioning**				
Brain fog	3%	(0–6%)	84%	(77–91%)
Unfocused concentration	4%	(0–8%)	81%	(73–89%)
Prioritizing multiple tasks	6%	(1–11%)	76%	(68–84%)
Multitasking	3%	(0–6%)	74%	(65–83%)
Mental apathy	4%	(0–8%)	72%	(63–81%)
Obsessive thoughts	4%	(0–8%)	56%	(46–66%)
Racing thoughts	1%	(0–3%)	54%	(44–64%)
Abstract reasoning	3%	(0–6%)	51%	(41–61%)
Intrusive thoughts	no data			
Time management	no data			
**Imagery**				
Vivid nightmares	3%	(0–6%)	38%	(28–48%)
Hypnagogic hallucinations	2%	(0–5%)	21%	(13–29%)
Illusions	2%	(0–5%)	20%	(12–28%)
Capacity for visual imagery	2%	(0–5%)	19%	(11–27%)
Intrusive aggressive images	1%	(0–3%)	19%	(11–27%)
Hallucinations (auditory, visual, olfactory, and tactile)	2%	(0–5%)	18%	(10–26%)
Intrusive images, other	1%	(0–3%)	10%	(4–16%)
Intrusive sexual images	1%	(0–3%)	6%	(1–11%)
**Emotional**				
Decreased frustration tolerance	5%	(1–9%)	80%	(72–88%)
Sudden mood swings	3%	(0–6%)	74%	(65–83%)
Anhedonia	3%	(0–6%)	64%	(55–73%)
Crying spells	0%	(0–0%)	50%	(40–60%)
Hypervigilance	1%	(0–3%)	45%	(35–55%)
Paranoia	1%	(0–3%)	26%	(17–35%)
Hyperarousal	no data			
**Dissociative symptoms**				
Depersonalization	2%	(0–5%)	64%	(55–73%)
Derealization	1%	(0–3%)	29%	(20–38%)
Dissociative Episodes	0%	(0–0%)	12%	(6–18%)
**Behavioral**				
Decreased job/school performance	2%	(0–5%)	78%	(70–86%)
Decreased social functioning	6%	(1–11%)	72%	(63–81%)
Compensatory compulsions	2%	(0–5%)	58%	(48–68%)
Dropping objects	2%	(0–5%)	52%	(42–62%)
Exaggerated startle reflex	1%	(0–3%)	49%	(39–59%)
Explosive anger	3%	(0–6%)	39%	(29–49%)
Marital/Family problems	4%	(0–8%)	39%	(29–49%)
Accident prone	4%	(0–8%)	35%	(26–44%)
Disinhibition	2%	(0–5%)	33%	(24–42%)
Suicidal	1%	(0–3%)	28%	(19–37%)
Substance abuse	1%	(0–3%)	12%	(6–18%)
Legal difficulties	1%	(0–3%)	8%	(3–13%)
Homicidal	0%	(0–0%)	1%	(0–3%)
**Psychiatric syndromes**				
Depression	9%	(3–15%)	79%	(71–87%)
Generalized anxiety disorder	3%	(0–6%)	53%	(43–63%)
Panic disorder	2%	(0–5%)	49%	(39–59%)
Social anxiety disorder	7%	(2–12%)	36%	(27–45%)
Obsessive compulsive disorder	2%	(0–5%)	24%	(16–32%)
Posttraumatic stress disorder	6%	(1–11%)	16%	(9–23%)
Rapid cycling bipolar	3%	(0–6%)	11%	(5–17%)
**Vegetative**				
**Energy**				
Fatigue	1%	(0–3%)	76%	(68–84%)
**Sleep**				
Non-restorative sleep	4%	(0–8%)	76%	(68–84%)
**Insomnia**				
Hypersomnia	2%	(0–5%)	73%	(64–82%)
Insomnia, mid	1%	(0–3%)	72%	(63–81%)
Insomnia, initial	5%	(1–9%)	70%	(61–79%)
Insomnia, late	1%	(0–3%)	58%	(48–68%)
Loss of circadian rhythm	5%	(1–9%)	44%	(34–54%)
Delayed sleep phase disorder	no data			
Sleep apnea, central	no data			
Sleep apnea, obstructive	no data			
Sleep paralysis	no data			
Cataplexy	no data			
Narcolepsy	no data			
**Eating**				
Anorexia	1%	(0–3%)	45%	(35–55%)
Weight loss	1%	(0–3%)	45%	(35–55%)
Non-appetite over-eating	2%	(0–5%)	34%	(25–43%)
Weight gain without increased food intake	1%	(0–3%)	27%	(18–36%)
Weight gain with increased food intake	2%	(0–5%)	22%	(14–30%)
**Sexual functioning**				
Decreased libido	4%	(0–8%)	60%	(50–70%)
Decreased arousal	1%	(0–3%)	42%	(32–52%)
Decreased orgasm	2%	(0–5%)	41%	(31–51%)
Increased libido	1%	(0–3%)	9%	(3–15%)
Altered sexual imagery	0%	(0–0%)	3%	(0–6%)
**Temperature control**				
Intolerance to cold	2%	(0–5%)	64%	(55–73%)
Body temperature fluctuations	3%	(0–6%)	63%	(54–72%)
Night sweats	2%	(0–5%)	60%	(50–70%)
Chills	2%	(0–5%)	59%	(49–69%)
Intolerance to heat	2%	(0–5%)	58%	(48–68%)
Decreased body temperature	5%	(1–9%)	52%	(42–62%)
Flushing	3%	(0–6%)	49%	(39–59%)
Low grade fevers	1%	(0–3%)	47%	(37–57%)
**Neurological**				
**Headache (neurological and musculoskeletal)**				
Headache	3%	(0–6%)	68%	(59–77%)
Tension	2%	(0–5%)	57%	(47–67%)
Cervical radiculopathy	0%	(0–0%)	43%	(33–53%)
Temporal mandibular joint	2%	(0–5%)	41%	(31–51%)
Sinus	5%	(1–9%)	41%	(31–51%)
Migraine	4%	(0–8%)	33%	(24–42%)
Cluster	0%	(0–0%)	10%	(4–16%)
Coital cephalgia	0%	(0–0%)	4%	(0–8%)
Thunderclap	no data			
**Cranial nerves**				
I Olfactory: loss of smell, altered taste	2%	(0–5%)	22%	(14–30%)
II Optic (and ophthalmologic)				
Photophobia to bright light	3%	(0–6%)	61%	(51–71%)
Floaters	1%	(0–3%)	56%	(46–66%)
Blurred vision	2%	(0–5%)	50%	(40–60%)
Sensitivity to fluorescent and flicker	3%	(0–6%)	48%	(38–58%)
Eye pain	2%	(0–5%)	36%	(27–45%)
Night blindness	4%	(0–8%)	36%	(27–45%)
Dry eyes	0%	(0–0%)	32%	(23–41%)
Flashes	0%	(0–0%)	23%	(15–31%)
Conjunctivitis	0%	(0–0%)	19%	((11–27%)
Peripheral shadows	2%	(0–5%)	18%	(18–26%)
Blind spots	1%	(0–3%)	12%	(6–18%)
Optic neuritis	0%	(0–0%)	2%	(0–5%)
Papilledema	0%	(0–0%)	1%	(0–3%)
Iritis	0%	(0–0%)	1%	(0–3%)
Panopsia	no data			
III, IV, VI Double vision or eye drifts when tired, ptosis	2%	(0–5%)	36%	(27–45%)
V Sensory loss, pain	0%	(0–0%)	27%	(18–36%)
VII Bell’s palsy	2%	(0–5%)	16%	(9–23%)
VIII Dizziness	2%	(0–5%)	53%	(43–63%)
Tinnitus	1%	(0–3%)	51%	(41–61%)
Motion sickness	9%	(3–15%)	40%	(30–50%)
Vertigo	1%	(0–3%)	29%	(20–38%)
Hearing loss	1%	(0–3%)	26%	(17–35%)
Tullio’s	0%	(0–0%)	12%	(6–18%)
Mal de debarquement	no data			
IX, X Episodic loss of speech, choking on food, difficulty swallowing	0%	(0–0%)	36%	(27–45%)
XI. Sternocleidomastoid and trapezius pain and/or paresis	0%	(0–0%)	44%	(34–54%)
XII. Tongue deviates to side	0%	(0–0%)	5%	(1–9%)
**Seizures**				
Partial	2%	(0–5%)	8%	(3–13%)
Grand mal	1%	(0–3%)	4%	(0–8%)
**Other neurological**				
Tingling	1%	(0–3%)	71%	(62–80%)
Paresis	2%	(0–5%)	66%	(57–75%)
Numbness	1%	(0–3%)	59%	(49–69%)
Twitching	1%	(0–3%)	56%	(46–66%)
Muscle tightness	0%	(0–0%)	56%	(46–66%)
Restless leg	5%	(1–9%)	50%	(40–60%)
Sensory loss	1%	(0–3%)	40%	(30–50%)
Tremor	3%	(0–6%)	40%	(30–50%)
Myoclonic jerks	1%	(0–3%)	38%	(28–48%)
Burning	1%	(0–3%)	36%	(27–45%)
Static electric sensation	0%	(0–0%)	35%	(26–44%)
Formication, crawling sensation	0%	(0–0%)	35%	(26–44%)
Stabbing sensation	0%	(0–0%)	28%	(19–37%)
Romberg positive	1%	(0–3%)	21%	(13–29%)
Herniated disc(s)	4%	(0–8%)	14%	(7–21%)
Ataxia	1%	(0–3%)	6%	(1–11%)
Other neurological	1%	(0–3%)	6%	(1–11%)
Extrapyramidal symptoms	0%	(0–0%)	3%	(0–6%)
Tourette’s	0%	(0–0%)	2%	(0–5%)
Torticollis	0%	(0–0%)	1%	(0–3%)
Spasticity	1%	(0–3%)	1%	(0–3%)
Sensation of wetness	no data			
Sensation of vibration	no data			
**Musculoskeletal**				
Joint pain, swelling, tightness, and crepitation (specify joints)	2%	(0–5%)	81%	(73–89%)
Myalgia	1%	(0–3%)	54%	(44–64%)
Chondritis (ear, nose, and costochondral)	0%	(0–0%)	38%	(28–48%)
Fibromyalgia	1%	(0–3%)	36%	(27–45%)
Plantar fasciitis	0%	(0–0%)	33%	(24–42%)
Epicondylitis	2%	(0–5%)	20%	(12–28%)
Tendonitis	3%	(0–6%)	17%	(10–24%)
Carpal tunnel	1%	(0–3%)	15%	(8–22%)
Bone thinning/fractures	1%	(0–3%)	7%	(2–12%)
Periostitis (tibia, ribs, iliac crest, sternum, clavicle, etc.	4%	(0–8%)	7%	(2–12%)
Deep bone pain	no data			
Foot pain	no data			
Ehlers-Danlos	no data			
**Cardiovascular**				
Racing pulse	0%	(0–0%)	48%	(38–58%)
Chest pain	2%	(0–5%)	39%	(29–49%)
Episodes rapid and slow heart rate	0%	(0–0%)	34%	(25–43%)
Mitral valve prolapse	4%	(0–8%)	20%	(12–28%)
Murmur	7%	(2–12%)	16%	(9–23%)
Hypertension	2%	(0–5%)	15%	(8–22%)
Postural orthostatic hypotension	0%	(0–0%)	12%	(6–18%)
Heart block	2%	(0–5%)	11%	(5–17%)
Hypertensive crisis	1%	(0–3%)	3%	(0–6%)
Cardiomyopathy	0%	(0–0%)	2%	(0–5%)
Pericarditis	0%	(0–0%)	1%	(0–3%)
Postural orthostatic tachycardia syndrome	no data			
**Upper respiratory, dental, and pulmonary**				
Shortness of breath	1%	(0–3%)	43%	(33–53%)
Swollen glands	0%	(0–0%)	41%	(31–51%)
Allergies	7%	(2–12%)	35%	(26–44%)
Tooth pain	0%	(0–0%)	32%	(23–41%)
Cough	1%	(0–3%)	28%	(19–37%)
Periodontal disease	0%	(0–0%)	19%	(11–27%)
Asthma	4%	(0–8%)	14%	(7–21%)
Nose bleeds	1%	(0–3%)	7%	(2–12%)
Air hunger	no data			
**Gastrointestinal**				
Irritable bowel	6%	(1–11%)	50%	(40–60%)
Abdominal bloating	1%	(0–3%)	42%	(32–52%)
Upper GI distress	6%	(1–11%)	25%	(17–33%)
Inflammatory bowel	0%	(0–0%)	2%	(0–5%)
Cholecystitis	0%	(0–0%)	2%	(0–5%)
Gastroparesis	0%	(0–0%)	1%	(0–3%)
Hepatitis	0%	(0–0%)	1%	(0–3%)
Pancreatitis	0%	(0–0%)	1%	(0–3%)
Gall stones	0%	(0–0%)	1%	(0–3%)
Non-calculous cholecystitis	no data			
Cyclic vomiting	no data			
**Genitourinary**				
Spastic bladder	1%	(0–3%)	47%	(37–57%)
Menstrual irregularity	3%	(0–6%)	30%	(21–39%)
Genital pain	1%	(0–3%)	27%	(18–36%)
Breast tenderness, pain	1%	(0–3%)	24%	(16–32%)
Urinary incontinence	1%	(0–3%)	18%	(10–26%)
Recurrent UTI	1%	(0–3%)	11%	(5–17%)
Lactation	0%	(0–0%)	8%	(3–13%)
Anesthesia of genitalia	0%	(0–0%)	6%	(1–11%)
Atrophy of genitalia	0%	(0–0%)	3%	(0–6%)
Interstitial cystitis	0%	(0–0%)	1%	(0–3%)
**Other**				
Hair loss	2%	(0–5%)	47%	(37–57%)
Chronic pain	0%	(0–0%)	41%	(31–51%)
Alcohol intolerance	3%	(0–6%)	41%	(31–51%)
Ecchymosis	1%	(0–3%)	34%	(25–43%)
Multiple chemical sensitivity	2%	(0–5%)	25%	(17–33%)
Thyroid dysfunction	1%	(0–3%)	20%	(12–28%)
Hypoglycemia	2%	(0–5%)	20%	(12–28%)
Ankle edema	1%	(0–3%)	20%	(12–28%)
Adrenal insufficiency	0%	(0–0%)	10%	(4–16%)
Vasculitis	0%	(0–0%)	5%	(1–9%)
Wilson syndrome	0%	(0–0%)	4%	(0–8%)
Splenomegaly	0%	(0–0%)	4%	(0–8%)
Lymphocytoma	3%	(0–6%)	3%	(0–6%)
Acrodermatitis chronicum atrophicans	0%	(0–0%)	1%	(0–3%)
Erythema of palms and soles	0%	(0–0%)	0%	(0–0%)
Mold sensitivity	no data			
Bartonella tracks	no data			
**Symptom patterns**				
Progression of symptoms	0%	(0–0%)	86%	(79–93%)
Fluctuation of symptoms	0%	(0–0%)	82%	(74–90%)
Stress increased symptoms	0%	(0–0%)	77%	(69–85%)
Herxheimer reaction	0%	(0–0%)	73%	(64–82%)
Antibiotic reduce symptoms	0%	(0–0%)	72%	(63–81%)
A 28 day or longer symptom cycle	0%	(0–0%)	43%	(33–53%)

**Table 2:** Pre-infection prevalence of mental disorders in the patients studied compared to the prevalence of the same disorders in the 12 month National Comorbidity Replication Survey.

Psychiatric Syndromes	Pre-Infection	95% CI	National Comorbidity Survey
Depression	9.0%	(3–15%)	8.2%
Rapid cycling bipolar	3.0%	(0–6%)	2.6%
Panic disorder	2.0%	(0–5%)	2.7%
Obsessive compulsive disorder	2.0%	(0–5%)	1.0%
Social anxiety disorder	7.0%	(2–12%)	6.8%
Generalized anxiety disorder	3.0%	(0–6%)	3.1%
Posttraumatic stress disorder	6.0%	(1–11%)	3.5%
Explosive anger	3.0%	(0–6%)	2.6%

**Table 3:** Clinical findings where ≥50% of patients report the finding.

Clinical Impairment	Pre-Infection	95% CI	Post-Infection	95% CI
**Attention span**				
Sustained attention	7%	(2–12%)	84%	(77–91%)
Distracted by frustration	7%	(2–12%)	79%	(71–87%)
Allocation of attention	6%	(1–11%)	66%	(57–75%)
Hypersensitivity to sound	3%	(0–6%)	66%	(57–75%)
Hypersensitivity to light	2%	(0–5%)	63%	(54–72%)
**Memory**				
Working memory	3%	(0–6%)	78%	(70–86%)
Recent memory	5%	(1–9%)	77%	(69–85%)
**Memory retrieval**				
Words	3%	(0–6%)	70%	(62–78%)
Names	6%	(1–11%)	68%	(60–76%)
Numbers	3%	(0–6%)	52%	(43–61%)
**Processing**				
Fluency of speech	4%	(0–8%)	62%	(54–70%)
Reading comprehension	6%	(1–11%)	59%	(50–68%)
Spelling errors	8%	(3–13%)	56%	(47–65%)
Word substitution errors	5%	(1–9%)	55%	(46–64%)
Optic ataxia	1%	(0–3%)	51%	(42–60%)
Calculation	10%	(4–16%)	51%	(43–59%)
**Executive functioning**				
Brain fog	3%	(0–6%)	84%	(78–90%)
Unfocused concentration	4%	(0–8%)	81%	(75–87%)
Prioritizing multiple tasks	6%	(1–11%)	76%	(69–83%)
Multitasking	3%	(0–6%)	74%	(67–81%)
Mental apathy	4%	(0–8%)	72%	(65–79%)
Obsessive thoughts	4%	(0–8%)	56%	(48–64%)
Racing thoughts	1%	(0–3%)	54%	(46–62%)
Abstract reasoning	3%	(0–6%)	51%	(43–59%)
**Emotional**				
Decreased frustration tolerance	5%	(1–9%)	80%	(74–86%)
Sudden mood swings	3%	(0–6%)	74%	(67–81%)
Anhedonia	3%	(0–6%)	64%	(57–71%)
Crying spells	0%	(0–0%)	50%	(42–58%)
**Dissociative symptoms**				
Depersonalization	2%	(0–5%)	64%	(57–71%)
**Behavioral**				
Decreased job/school performance	2%	(0–5%)	78%	(72–84%)
Decreased social functioning	6%	(1–11%)	72%	(65–79%)
Compensatory compulsions	2%	(0–5%)	58%	(51–65%)
Dropping objects	2%	(0–5%)	52%	(45–59%)
**Psychiatric syndromes**				
Depression	9%	(3–15%)	79%	(73–85%)
Generalized anxiety disorder	3%	(0–6%)	53%	(46–60%)
**Vegetative**				
**Energy**				
Fatigue	1%	(0–3%)	76%	(70–82%)
**Sleep**				
Non-restorative sleep	4%	(0–8%)	76%	(70–82%)
**Insomnia**				
Hypersomnia	2%	(0–5%)	73%	(67–79%)
Insomnia, mid	1%	(0–3%)	72%	(66–78%)
Insomnia, initial	5%	(1–9%)	70%	(64–76%)
Insomnia, late	1%	(0–3%)	58%	(51–65%)
Loss of circadian rhythm	5%	(1–9%)	44%	(37–51%)
**Sexual functioning**				
Decreased libido	4%	(0–8%)	60%	(54–66%)
**Temperature control**				
Intolerance to cold	2%	(0–5%)	64%	(58–70%)
Body temperature fluctuations	3%	(0–6%)	63%	(57–69%)
Night sweats	2%	(0–5%)	60%	(54–66%)
Intolerance to heat	2%	(0–5%)	58%	(52–64%)
Decreased body temperature	5%	(1–9%)	52%	(46–58%)
Chills	2%	(0–5%)	59%	(53–65%)
**Neurological**				
Headache	3%	(0–6%)	68%	(62–74%)
Tension headache	2%	(0–5%)	57%	(51–63%)
**Cranial nerves**				
II Optic (and ophthalmologic)				
Photophobia to bright light	3%	(0–6%)	61%	(55–67%)
Floaters	1%	(0–3%)	56%	(50–62%)
Dizziness	2%	(0–5%)	53%	(47–59%)
VIII Tinnitus	1%	(0–3%)	51%	(45–57%)
Blurred vision	2%	(0–5%)	50%	(44–56%)
**Other neurological**				
Numbness	1%	(0–3%)	59%	(53–65%)
Tingling	1%	(0–3%)	71%	(66–76%)
Paresis	2%	(0–5%)	66%	(61–71%)
Tremor	3%	(0–6%)	40%	(34–46%)
Twitching	1%	(0–3%)	56%	(50–62%)
Muscle tightness	0%	(0–0%)	56%	(50–62%)
Restless leg	5%	(1–9%)	50%	(44–56%)
**Musculoskeletal**				
Joint pain, swelling, tightness, and crepitation (specify joints)	2%	(0–5%)	81%	(77–85%)
Myalgia	1%	(0–3%)	54%	(49–59%)
**Gastrointestinal**				
Irritable bowel	6%	(1–11%)	50%	(45–55%)
**Symptom pattern**				
Progression of symptoms	0%	(0–0%)	86%	(79–93%)
Fluctuation of symptoms	0%	(0–0%)	82%	(74–90%)
Stress increased symptoms	0%	(0–0%)	77%	(69–85%)
Herxheimer reaction	0%	(0–0%)	73%	(64–82%)
Antibiotic reduce symptoms	0%	(0–0%)	72%	(63–81%)

**Table 4:** Post-infection findings compared to the prevalence of the same findings in other studies.

Psychiatric Syndromes	Post-Infection	Other Lyme Patient Studies (Reference [9])
Sustained attention	84%	(44%, 91%)
Distracted by frustration	79%	(82%)
Allocation of attention	66%	(98%)
Hypersensitivity to sound	66%	(58%, 88%)
Hypersensitivity to light	63%	(74%)
Working memory	78%	(98%)
Recent memory	77%	(94%)
Fluency of speech	62%	(46%, 75%, 79%, 82%)
Reading comprehension	59%	(79%)
Auditory comprehension	49%	(73%)
Brain fog	84%	(88%)
Abstract reasoning impairments	51%	(60%, 93%)
Vivid nightmares	38%	(58%, 70%, 82%)
Intrusive aggressive images	19%	(16%, 62%),
Intrusive sexual images	6%	(26%, 16%, 6%)
Hallucinations	18%	(42%, 45%, 47%)
Decreased frustration tolerance	80%	(80%, 98%)
Sudden mood swings	74%	(15%, 47%, 66%, 85%, 93%, 94%)
Anhedonia	64%	(56%, 59%, 71%, 72%, 85%)
Exaggerated startle reflex	49%	(66%, 75%, 84%)
Hypervigilance	45%	(35%, 54%, 55%, 69%, 72%, 84%)
Disinhibition	33%	(20, 32%, 35%, 58%, 80%, 84%)
Paranoia	26%	(10%, 25%, 36%, 62%, 76%, 88%)
Dissociative episodes	12%	(0%, 5%, 12%, 18%, 25%, 38%)
Dysphoria/depression	79%	(37%, 37%, 50%, 51%, 64%, 70%, 76%, 80%, 97%, 98%, 100%)
Generalized anxiety disorder	55%	(50%, 65%, 70%, 86%, 90%)
Panic disorder	49%	(35%, 50%, 54%, 80%, 82%)
Social anxiety disorder	36%	(20%, 55%, 65%, 66%, 68%, 70%)
Obsessive compulsive disorder	24%	(32%, 42%, 44%, 51%, 84%)
Posttraumatic stress disorder	16%	(15%, 15%, 24%, 30%, 36%)
Rapid cycling bipolar	11%	(5%, 10%, 19%, 20%, 21%, 28%)
Depersonalization	64%	(40%, 52%, 55%, 71%, 76%)
Derealization	29%	(24%, 31%, 37%)
Decreased school/job performance	78%	(94%)
Decreased social functioning	72%	(91%)
Explosive anger	39%	(52%, 72%, 91%)
Marital/family problems	39%	(48%, 80%)
Suicidal	28%	(20%, 43%, 46%, 63% 72%, 98%)
Substance abuse	12%	(5%, 10%, 10%, 28%, 33%)
Legal problems	8%	(4%, 42%)
Homicidal	1%	(9.6%)
Fatigue	73%	(85%, 85%, 92%, 97%)
Irritable bladder	47%	(44%, 50%, 56%)
Genital pain	26%	(24%, 28%, 32%)
Decreased libido	22%	(38%, 44%, 62%, 80%)
Urinary incontinence	18%	(18%, 28%, 38%)
Chronic pain	41%	(35%, 57%, 65%)
Alcohol intolerance	11%	(24%, 34%, 44%)

**Table 5:** Common symptom assessment in which ≥50% or more have the clinical finding.

**Name:**			**Birthdate:**			
**Date of infection:**			**Date of diagnosis:**			
**Date of initial antibiotic treatment:**						
**Exposure to endemic area:**						
**History of tick bites:**						
**History of erythema migrans rash:**						
**Flu-like symptoms:**						
**Recurrent erythema migrans rash:**						
**Laboratory findings:**						
**CDC surveillance criteria**						
**Co-infections:**						
**Pre-existing conditions:**						
**Prior diagnosis:**						
**Clinical Impairment**	**Database: Pre-infection**	**Database: Post-infection**	**Patient: Pre-infection**	**Patient: Anytime post-infection**	**Patient: Initial assessment**	**Patient: Subsequent assessment**
	The presence of a pathological clinical finding: enter as (+)					
	The absence of a pathological clinical finding: enter as (−)					
	The presence of a partial or episodic pathological clinical finding: enter as (+/−)					
**Attention span**						
Sustained attention	7%	84%				
Distracted by frustration	7%	79%				
Allocation of attention	6%	66%				
Hypersensitivity to sound	3%	66%				
Hypersensitivity to light	2%	63%				
**Memory**						
Working memory	3%	78%				
Recent memory	5%	77%				
Remote memory	4%	35%				
**Memory retrieval**						
Words	3%	70%				
Names	6%	68%				
Numbers	3%	52%				
**Processing**						
Fluency of speech	4%	62%				
Reading comprehension	6%	59%				
Spelling errors	8%	56%				
Word substitution errors	5%	55%				
Optic ataxia	1%	51%				
Calculation	10%	51%				
**Executive functioning**						
Brain fog	3%	84%				
Unfocused concentration	4%	81%				
Prioritizing multiple tasks	6%	76%				
Multitasking	3%	74%				
Mental apathy	4%	72%				
**Emotional**						
Decreased frustration tolerance	5%	80%				
Sudden mood swings	3%	74%				
**Behavioral**						
Decreased job/school performance	2%	78%				
Decreased social functioning	6%	72%				
Dropping objects	2%	52%				
**Psychiatric syndromes**						
Depression	9%	79%				
Generalized anxiety disorder	3%	53%				
Posttraumatic stress disorder	6%	16%				
**Vegetative**						
**Energy**						
Fatigue	1%	76%				
**Sleep**						
Non-restorative sleep	4%	76%				
**Insomnia**						
Hypersomnia	2%	73%				
Insomnia, mid	1%	72%				
Insomnia, initial	5%	70%				
Insomnia, late	1%	58%				
**Sexual functioning**						
Decreased libido	4%	60%				
**Temperature control**						
Intolerance to cold	2%	64%				
Body temperature fluctuations	3%	63%				
Night sweats	2%	60%				
Chills	2%	59%				
Intolerance to heat	2%	58%				
Decreased body temperature	5%	52%				
**Neurological**						
Headache (neurological & other)	3%	68%				
Tension headache	2%	57%				
**Cranial nerves**						
II Optic/ophthalmologic						
Photophobia to bright light	3%	61%				
Floaters	1%	56%				
Dizziness	2%	53%				
VIII Tinnitus	1%	51%				
Blurred vision	2%	50%				
**Other neurological**						
Tingling	1%	71%				
Paresis	2%	66%				
Numbness	1%	59%				
Twitching	1%	56%				
Muscle tightness	0%	56%				
**Musculoskeletal**						
Joint pain, swelling, tightness, and crepitation (specify joints)	2%	81%				
Myalgia	1%	54%				
**Gastrointestinal**						
Irritable bowel	6%	50%				
**Symptom patterns**						
Progression of symptoms	0%	86%				
Fluctuation of symptoms	0%	82%				
Stress increased symptoms	0%	77%				
Herxheimer reaction	0%	73%				
Antibiotic reduce symptoms	0%	72%				
A 28 day or longer symptom cycle	0%	43%				

**Table 6:** Reduced set of 24 highly significant impairments. Suitable for pre-evaluation by patients.

**Name:**		**Birthdate:**		
**Date of symptoms onset:**				
**Exposure to endemic area:**				
**History of tick bites:**				
**History of erythema migrans rash:**				
**Flu-like symptoms:**				
**Recurrent erythema migrans rash:**				
**Pre-existing conditions:**				
**Clinical impairment** **(check if symptom present)**	**Prior to illness**	**Symptom since** **illness began**	**Current symptom**	**Follow up**
Concentration impairment				
Short term memory problems				
Word finding difficulty				
Name recall difficulty				
Fluency of speech difficulties				
Brain fog				
Sudden mood swings				
Decreased social functioning				
Decreased job/school performance				
Depression				
Fatigue				
Insomnia				
Night sweats				
Low body temperature				
Headache				
Blurred vision				
Floaters				
Tinnitus (ringing in the ears)				
Sensitive to sound				
Dizziness				
Numbness				
Tingling				
Joint pain, swelling				
Fluctuation of symptoms				
Stress increases symptoms				

**Table 7:** Full assessment.

**Name:**				**Birthdate:**		
**Date of infection:**				**Date of diagnosis:**		
**Date of initial antibiotic treatment:**						
**Exposure to endemic area:**						
**History of tick bites:**						
**History of erythema migrans rash:**						
**Flu-like symptoms:**						
**Recurrent erythema migrans rash:**						
**Laboratory findings:**						
**CDC surveillance criteria:**						
**Co-infections:**						
**Pre-existing conditions:**						
**Prior diagnosis:**						
	+ present	− absent	+/− partial	+/− -slight	or CGI-S	or CGI-I
**Clinical impairment**	**Database: Pre-infection**	**Database: Post-infection**	**Patient: Pre-infection**	**Patient: Anytime post-infection**	**Patient: Current Symptom**	**Patient: Follow up**
**Attention span**						
Sustained attention	7%	84%				
Distracted by frustration	7%	79%				
Allocation of attention	6%	66%				
Hypersensitivity to sound	3%	66%				
Hypersensitivity to light	2%	63%				
Hypersensitivity to touch	2%	41%				
Hypersensitivity to smell	5%	36%				
Sensory overload	no data					
**Memory impairments**						
Working memory	3%	78%				
Recent memory	5%	77%				
Working spatial memory	1%	46%				
Remote memory	4%	35%				
**Memory retrieval difficulties**						
Words	3%	70%				
Names	6%	68%				
Number	3%	52%				
Geographical	1%	49%				
Faces	1%	23%				
Motor memory	1%	10%				
**Processing**						
Fluency of speech	4%	62%				
Reading comprehension	6%	59%				
Spelling errors	8%	56%				
Word substitution errors	5%	55%				
Calculation	10%	51%				
Optic ataxia	1%	51%				
Auditory comprehension	5%	49%				
Handwriting	8%	47%				
Letter reversal	2%	45%				
Fluency of written language	2%	43%				
Number reversal	1%	39%				
Left–right confusion	6%	30%				
Transposition of laterality	2%	22%				
Spatial perceptual distortions	1%	21%				
Sound localization	3%	19%				
Slow processing	no data					
**Executive functioning**						
Brain fog	3%	84%				
Unfocused concentration	4%	81%				
Prioritizing multiple tasks	6%	76%				
Multitasking	3%	74%				
Mental apathy	4%	72%				
Obsessive thoughts	4%	56%				
Racing thoughts	1%	54%				
Abstract reasoning	3%	51%				
Intrusive thoughts	no data					
Time management	no data					
**Imagery**						
Vivid nightmares	3%	38%				
Hypnagogic hallucinations	2%	21%				
Illusions (auditory, visual	2%	20%				
Capacity for visual imagery	2%	19%				
Hallucinations (auditory, musical, visual, olfactory, and tactile)	2%	18%				
Intrusive aggressive images	1%	19%				
Intrusive sexual images	1%	6%				
Intrusive images, other	1%	10%				
**Emotional**						
Decreased frustration tolerance	5%	80%				
Sudden mood swings	3%	74%				
Anhedonia	3%	64%				
Crying spells	0%	50%				
Hypervigilance	1%	45%				
Paranoia	1%	26%				
Hyperarousal	no data					
Intrusive emotions	no data					
Other						
**Dissociative symptoms**						
Depersonalization	2%	64%				
Derealization	1%	29%				
Dissociative episodes	0%	12%				
**Behavioral**						
Decreased job/school performance	2%	78%				
Decreased social functioning	6%	72%				
Compensatory compulsions	2%	58%				
Dropping objects	2%	52%				
Exaggerated startle reflex	1%	49%				
Marital/family problems	4%	39%				
Explosive anger	3%	39%				
Accident prone	4%	35%				
Disinhibition	2%	33%				
Suicidal	1%	28%				
Substance abuse	1%	12%				
Legal difficulties	1%	8%				
Homicidal	0%	1%				
Other						
**Psychiatric syndromes**						
Depression	9%	79%				
Generalized anxiety disorder	3%	53%				
Panic disorder	2%	49%				
Social anxiety disorder	7%	36%				
Obsessive compulsive disorder	2%	24%				
Posttraumatic stress disorder	6%	16%				
Rapid cycling bipolar	36%	11%				
Grooming disorder	no data					
Other						
**Vegetative**						
**Energy**						
Fatigue	1%	76%				
**Sleep**						
Non-restorative sleep	4%	76%				
**Insomnia**						
Insomnia, initial	5%	70%				
Insomnia, mid	1%	72%				
Insomnia, late	1%	58%				
Hypersomnia	2%	73%				
Loss of circadian rhythm	5%	44%				
Delayed sleep phase disorder	no data					
Sleep apnea, central	no data					
Sleep apnea, obstructive	no data					
Sleep paralysis	no data					
Cataplexy	no data					
Narcolepsy	no data					
**Eating**						
Anorexia	1%	45%				
Weight loss	1%	45%				
Non-appetite over-eating	2%	34%				
Weight gain without increased food intake	1%	27%				
Weight gain with increased food intake	2%	22%				
**Sexual functioning**						
Decreased libido	4%	60%				
Decreased arousal	1%	42%				
Decreased orgasm	2%	41%				
Increased libido	1%	9%				
Altered sexual imagery	0%	3%				
**Temperature control**						
Intolerance to cold	2%	64%				
Body temperature fluctuations	3%	63%				
Night sweats	2%	60%				
Chills	2%	59%				
Intolerance to heat	2%	58%				
Decreased body temperature	5%	52%				
Flushing	3%	49%				
Low grade fevers	1%	47%				
**Neurological**						
**Headache (neurological, musculoskeletal, & other)**						
Headache	3%	68%				
Tension	2%	57%				
Cervical radiculopathy	0%	43%				
Temporal mandibular joint	2%	41%				
Sinus	5%	41%				
Migraine	4%	33%				
Cluster	0%	10%				
Coital cephalgia	0%	4%				
Thunderclap	no data					
Other						
**Cranial nerves**						
I Olfactory: loss of smell, altered taste	2%	22%				
II Optic & ophthalmologic						
Photophobia to bright light	3%	61%				
Floaters	1%	56%				
Blurred vision	2%	50%				
Sensitivity to fluorescent and flicker	3%	48%				
Night blindness	4%	36%				
Eye pain	2%	36%				
Dry eyes	0%	32%				
Flashes	0%	23%				
Conjunctivitis	0%	19%				
Peripheral shadows	2%	18%				
Blind spots	1%	12%				
Optic neuritis	0%	2%				
Iritis	0%	1%				
Uveitis	0%	1%				
Papilledema	0%	1%				
Panopsia	no data					
III, IV, VI Double vision or eye drifts when tired, ptosis	2%	36%				
V Sensory loss, pain	0%	27%				
VII Bell’s palsy	2%	16%				
VIII Dizziness	2%	53%				
Tinnitus	1%	51%				
Motion sickness	9%	40%				
Vertigo	1%	29%				
Hearing loss	1%	26%				
Tullio’s	0%	12%				
Mal de debarquement	no data					
IX, X Episodic loss of speech, choking on food, difficulty swallowing	0%	36%				
XI. Sternocleidomastoid and trapezius pain and paresis	0%	44%				
XII. Tongue deviates to side	0%	5%				
**Seizures**						
Partial	2%	8%				
Grand mal	1%	4%				
**Other neurological**						
Tingling	1%	71%				
Numbness	1%	59%				
Sensory loss	1%	40%				
Burning	1%	36%				
Static electric sensation	0%	35%				
Formication, crawling sensation	0%	35%				
Stabbing sensation	0%	28%				
Sensation of wetness	no data					
Sensation of vibration	no data					
Paresis	2%	66%				
Muscle tightness	0%	56%				
Twitching	1%	56%				
Restless leg	5%	50%				
Tremor	3%	40%				
Myoclonic jerks	1%	38%				
Ataxia	1%	6%				
Extrapyramidal symptoms	0%	3%				
Tourette’s	0%	2%				
Torticollis	0%	1%				
Herniated disc(s)	4%	14%				
Other neurological	1%	6%				
Romberg	1%	21%				
**Musculoskeletal**						
Joint pain, swelling, tightness, and crepitation (specify joints) (migratory?)	2%	81%				
Myalgia	1%	54%				
Chondritis (ear, nose, and costochondral)	0%	38%				
Fibromyalgia	1%	36%				
Plantar fasciitis	0%	33%				
Epicondylitis	2%	20%				
Tendonitis	3%	17%				
Carpal tunnel	1%	15%				
Periostitis (tibia, ribs, iliac crest, sternum, clavicle, etc.)	4%	7%				
Bone thinning/fractures	1%	7%				
Deep bone pain	no data					
Foot pain	no data					
Ehlers-Danlos	no data					
**Cardiovascular**						
Racing pulse	0%	48%				
Chest pain	2%	39%				
Episodes rapid and slow heart rate	0%	34%				
Mitral valve prolapse	4%	20%				
Murmur	7%	16%				
Hypertension	2%	15%				
Hypertensive crisis	1%	3%				
Postural orthostatic hypotension	0%	12%				
Postural orthostatic tachycardia	no data					
Heart block	2%	11%				
Cardiomyopathy	0%	2%				
Pericarditis	0%	1%				
**Upper respiratory, dental, and pulmonary**						
Shortness of breath	1%	43%				
Air hunger	no data					
Swollen glands	0%	41%				
Allergies	7%	35%				
Tooth pain	0%	32%				
Periodontal disease	0%	19%				
Cough	1%	28%				
Asthma	4%	14%				
Nose bleeds	1%	7%				
**Gastrointestinal**						
Irritable bowel	6%	50%				
Abdominal bloating	1%	42%				
Upper GI distress	6%	25%				
Inflammatory bowel	0%	2%				
Cholecystitis	0%	2%				
Non-calculous cholecystitis	no data					
Gall stones	0%	1%				
Hepatitis	0%	1%				
Pancreatitis	0%	1%				
Gastroparesis	0%	1%				
Cyclic vomiting	no data					
**Genitourinary**						
Spastic bladder	1%	47%				
Genital pain	1%	27%				
Urinary incontinence	1%	18%				
Recurrent urinary tract infection	1%	11%				
Anesthesia of genitalia	0%	6%				
Atrophy of genitalia	1%	3%				
Menstrual irregularity	3%	30%				
Breast tenderness, pain	1%	24%				
Lactation	0%	8%				
Interstitial cystitis	0%	1%				
**Other**						
Hair loss	2%	47%				
Chronic pain	0%	41%				
Alcohol intolerance	3%	41%				
Ecchymoses	1%	34%				
Multiple chemical sensitivity	2%	25%				
Hypoglycemia	2%	20%				
Ankle edema	1%	20%				
Thyroid dysfunction	1%	20%				
Wilson syndrome	0%	4%				
Adrenal insufficiency	0%	10%				
Vasculitis	0%	5%				
Splenomegaly	0%	4%				
Lymphocytoma	3%	3%				
Acrodermatitis chronicum atrophicans	0%	1%				
Bartonella tracks	no data					
Mold sensitivity	no data					
Other						
**Symptom patterns**						
Progression of symptoms	0%	86%				
Fluctuation of symptoms	0%	82%				
Stress increased symptoms	0%	77%				
Herxheimer reaction	0%	73%				
Antibiotic reduce symptoms	0%	72%				
A 28 day or longer symptom cycle	0%	43%				
**Prior treatment and response** Psychiatric treatments:						
Oral antibiotics:						
Intramuscular antibiotics:						
Intravenous antibiotics:						
Other treatments:						
Diagnosis:						
Prioritizing symptom contribution to disease progression and perpetuation:						
Treatment options:						
Risks vs. benefits & informed consent:						
Treatment plan:						
Treatment response:						

## Supplementary Materials

Materials are available online at https://www.mdpi.com/2227-9032/8/1/13/s1. Coinfection Screen: Sorting out Lyme and Associated Coinfections.
