# Supplementary material for: A Clinical Diagnostic System for Late-Stage Neuropsychiatric Lyme Borreliosis Based upon an Analysis of 100 Patients
Source: Healthcare (Basel). 2020 Jan 6;8(1):13. doi: 10.3390/healthcare8010013 (PMC7151210; doi:10.3390/healthcare8010013)
Supplement: Supplementary file 1 [file healthcare-08-00013-s001.pdf]

Coinfection Screen: Sorting out Lyme and Associated Coinfections

This assessment was written by Joseph J Burrascano JJ and was posted on *LymeDisease.org*. Available online: https://www.lymedisease.org/members/resources/lyme-disease-resources-non-members/ (accessed on 7 December 2019).

Classic Lyme (Bb infection)

Gradual onset of initial (viral-like) symptoms–this often makes it difficult to pinpoint when the infection began. Also, as in the case with Bb infection, laboratory tests for them are often insensitive. Thus, there is a need to sort it all out clinically to provide guidance in testing and treatment. Here are some clues:

- Multisystem–almost always, in disseminated stages, involves more than one part or system (i.e., joint pain plus cognitive dysfunction).
- Migratory–first a knee will hurt, then over time this may lessen, and the elbow or shoulder acts up, and later the joints calm down, but headaches worsen.
- Stiff joints and loud joint crepitus, especially the neck ("Lyme shrug").
- Headaches are often nuchal and associated with stiff, painful, and crepitant neck.
- Afternoon fevers, often unnoticed–most Lyme patients have subnormal temperatures in the morning but rise to 99+ by early to mid-afternoon. No obvious sweats.
- Tiredness and limited stamina- often is a strong need to rest or even nap in the afternoon, especially when the flushed face and elevated temperature appears.
- Four-week cycles–Bb activity, and thus symptoms, wax and wane in a cycle that repeats roughly every four weeks. This cycle, if clear, can guide your treatments.
- Slow response to treatment, with an initial symptom flare in most ("Herxheimer-like reaction"), then improvement over weeks, punctuated by the monthly symptom flares. Likewise, if treatment is ended too soon, an initial period of well-being will gradually be replaced by a return of symptoms over a few weeks.
- EM rash in 25% to 50% of patients.

Bartonella and "Bartonella-Like Organisms"

- Gradual onset of initial illness.
- Central nervous system symptoms are out of proportion to the musculoskeletal ones and can include muscle twitches, tremors, insomnia, seizures, agitation, anxiety, severe mood swings, outbursts, and antisocial behavior.
- Gastrointestinal involvement may present as gastritis or abdominal pain (mesenteric adenitis).
- Sore soles, especially in the morning.
- Tender subcutaneous nodules along the extremities, especially outer thigh, shins, and occasionally along the triceps.
- Occasional lymphadenopathy.
- Morning fevers, usually around 99; occasionally light sweats are noted.
- Elevated vascular endothelial growth factor (VEGF) occurs in a minority, but the degree of elevation correlates with activity of the infection and may be used to monitor treatment.
- Rapid response to treatment changes–often symptoms improve within days after antibiotics are begun, but relapses occur also within days if medication is withdrawn early.
- May have papular or linear red rashes (like stretch marks that do not always follow skin planes), especially in those with GI involvement.

Babesia Species

- Rapid onset of initial illness, often with sudden onset of high fever, severe headaches, sweats, and fatigue; thus, it is easy to know when infection began.
- Obvious sweats, usually at night, but can be day sweats as well.
- Air hunger, the need to sigh and take a deep breath; dry cough without apparent reason.
- Headaches can be severe–dull, global (involves the whole head, described like the head is in a vise).
- Fatigue is prominent, does not clear with rest, and is made worse with exercise.
- Mental dullness and slowing of reactions and responses.
- Dizziness–more like a tippy feeling, and not vertigo or purely orthostasis.
- Symptoms cycle rapidly, with flares every four to six days.
- Hypercoagulation is often associated with Babesia infections.
- Rarely, splenomegaly.
- Very severe Lyme disease can be a clue to Babesia infection, as it will make Lyme symptoms worse and Lyme treatments less effective.

Ehrlichia/Anaplasma

- Rapid onset of initial illness with fever, headache, prostration.
- Headaches are sharp, knife-like, and often behind the eyes.
- Muscle pain, not joint pain, and can be mild or severe.
- Low WBC, low platelet count, elevated liver enzymes, and (rarely) inclusions seen in the WBCs.
- Rarely see diffuse vasculitic rash, including palms and soles (less than 10%).
- Rapid response to treatment.
-

DNA Viruses (HHV-6, EBV, CMV)

- Persistent fatigue, made worse with exercise.
- Sore throat, lymphadenopathy, and other viral-like complaints.
- May see elevated liver enzymes and low WBCs.

Mycoplasma

- Gradual onset.
- May be light night sweats.
- Symptoms are made worse with exercise.
- Major fatigue and neurological dysfunction, especially autonomic neuropathies.
- Metabolic disturbances, immune damage, very low CD57 count (less than 20).
- Found in the sickest and most poorly responding Lyme patients (CFIDS-like).
